# Evaluation of the effects of fenestration in Fontan circulation using a lumped parameter model

**DOI:** 10.1186/s12576-024-00947-y

**Published:** 2024-12-21

**Authors:** Naohiro Horio, Shuji Shimizu, Yasuhiro Kotani, Yoshinori Miyahara, Shingo Kasahara

**Affiliations:** 1 Pediatric Heart Disease and Adult Congenital Heart Disease Center, Showa University Hospital, Tokyo, Japan; 2 Department of Cardiovascular Surgery, Okayama University Graduate School of Medicine, Dentistry and Pharmaceutical Sciences, Okayama, Japan; 3 Department of Research Promotion and Management, National Cerebral and Cardiovascular Center, 6-1 Kishibe-Shimmachi, Suita, Osaka 5648565 Japan

**Keywords:** Single ventricle, Fontan circulation, Fenestration, Hemodynamic simulation, Lumped parameter model

## Abstract

Fenestration has been reported to enhance Fontan hemodynamics in several cases of Fontan circulation. However, the indication criteria for fenestration remain under discussion. To assess the effectiveness of fenestration in Fontan circulation, we conducted a theoretical analysis using a computational model of the fenestrated Fontan circulation. The cardiac chambers and vascular systems were modeled using the time-varying elastance model and the modified Windkessel model, respectively. When the pulmonary vascular resistance index was 4.01 Wood units m^2^, fenestration significantly reduced central venous pressure from 18.0 to 16.1 mmHg and decreased stressed blood volume from 610 to 555 ml. However, in the models with reduced ventricular end-systolic elastance, increased ventricular stiffness constant, or heightened systemic vascular resistance, the advantages of fenestration were diminished. Thus, fenestration may effectively improve the hemodynamics of Fontan circulation in patients with elevated pulmonary vascular resistance.

## Introduction

**Fig. 1:**
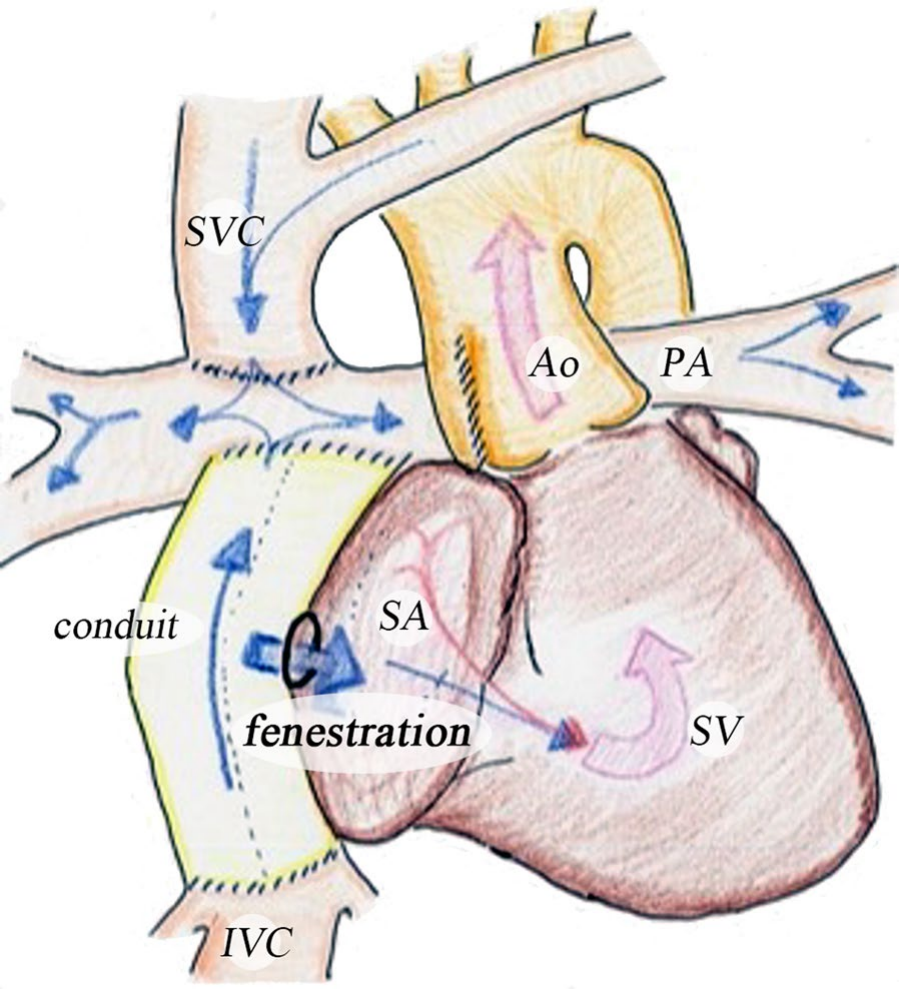
A schema of the Fontan operation with fenestration. *SV* single ventricle, *SA* single atrium, *Ao* aorta, *PA* pulmonary artery, *SVC* superior vena cava, *IVC* inferior vena cava

The Fontan procedure, first described by Francis Fontan in 1971, is a palliative surgery for patients with functional single ventricle [1]. The outcomes of the Fontan operation have been improved due to a staged palliation strategy and various modifications. Fenestration is one of these modifications [2–5]. Introduced in 1989, fenestration creates an opening between the conduit of the total cavopulmonary connection and the single atrium (Fig. 1), targeting high-risk Fontan candidates [6]. The benefits of fenestration are believed to include reduced systemic venous pressure and increased cardiac output by increasing preload to the systemic single ventricle [6–8]. Several studies have documented the advantages of fenestration in the early postoperative period, such as reduced pleural drainage, shorter hospital stays, and improved short-term morbidity [6, 9]. However, in the Fontan circulation, fenestration can lead to desaturation and increase the risk of thromboembolism. Several studies have reported no clinical benefits from fenestration [7]. As a result, the decision to use fenestration remains controversial and depends on the surgeon’s experience or institutional policy.

With advances in computer technology, hemodynamic simulations of congenital heart diseases have become more accessible. It is now possible to perform hemodynamic simulations using a lumped parameter model on a standard desktop computer. This method could provide invaluable for clinical decision making [10]. Various researchers have developed models for Fontan circulation. Liang et al. created models for normal and Fontan circulations model and compared their hemodynamic performance under different cardiovascular conditions [11]. Kung et al. developed a model to simulate exercise physiology in Fontan patients [12]. Meanwhile, Shimizu et al. explored the hemodynamic effects of a partial cavopulmonary assistance in failing Fontan patients [13]. However, the simulation of fenestrated Fontan circulation has been limited in previous studies [14, 15]. In our study, we investigated the efficacy of fenestration using computational simulations based on the lumped parameter model. Our goal was to identify the specific circumstances under which fenestration should be considered as an addition to the Fontan operation.

## Materials and methods

**Fig. 2:**
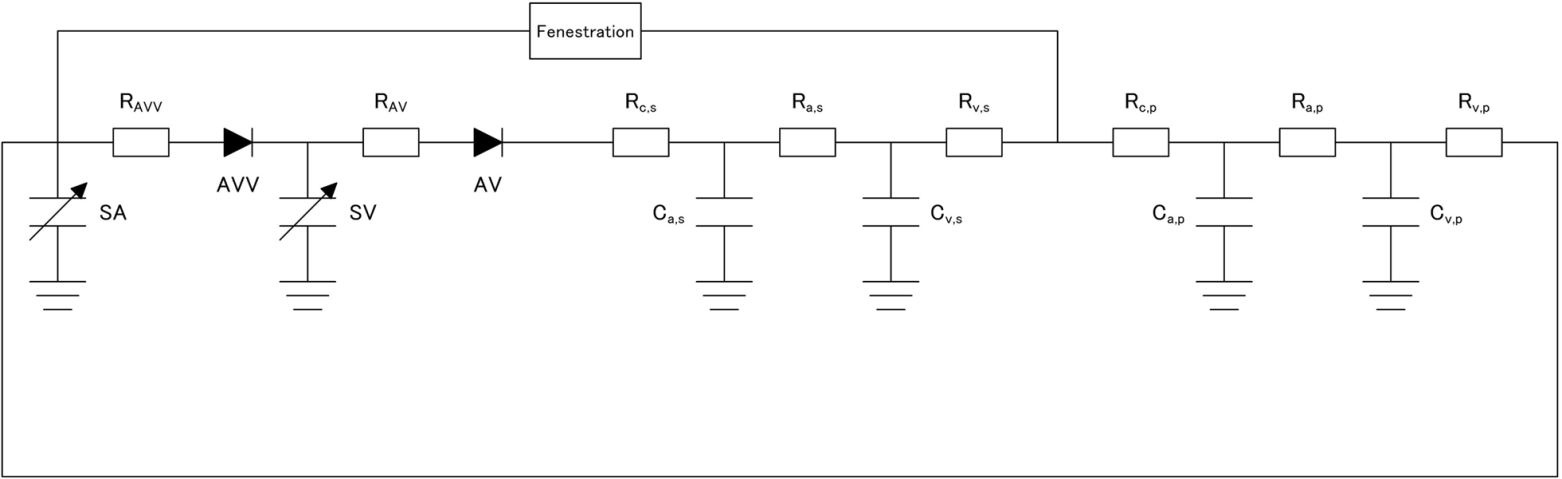
An electrical analog of the Fontan circulation. *SV* single ventricle, *SA* single atrium, *AVV* atrioventricular valve, *AV* aortic valve, *C*_*a*_* and C*_*v*_ lumped arterial and venous capacitances, *R*_*c*_ characteristic impedances, *R*_*a*_ lumped arterial resistance, *R*_*v*_ venous resistances, *s* systemic circulation, *p* pulmonary circulation

We modeled the cardiovascular systems of fenestrated Fontan circulation using a lumped parameter model [10]. This model is grounded in the time-varying elastance cardiac chamber model and the modified Windkessel model for vasculature. An electrical analog which simulates the cardiovascular systems of fenestrated Fontan circulation, is depicted in Fig. 2.

### Heart

The ventricular and atrial chambers are represented by the time-varying elastance model [16]. The end-systolic and end-diastolic pressure–volume relationships are described below:

1 $${\text{P}}_{\text{es},\text{cc}}\left(\text{t}\right)={\text{E}}_{\text{es},\text{cc}}\left({\text{V}}_{\text{es},\text{cc}}\left(\text{t}\right)-{\text{V}}_{0,\text{cc}}\right)$$

2 $${P}_{ed,cc}\left(t\right)={A}_{cc}\left[{e}^{{B}_{cc}\left({V}_{ed,cc}\left(t\right)-{V}_{0,cc}\right)}-1\right]$$

where P_es,cc_ is end-systolic pressure, V_es,cc_ is end-systolic volume, E_es,cc_ is the end-systolic elastance, V_0,cc_ is the volume at which end-systolic pressure is equal to 0 mmHg, P_ed,cc_ is end-diastolic pressure, V_ed,cc_ is end-diastolic volume, A_cc_ and B_cc_ are constants and cc denotes each chamber, i.e., SA for the single atrium and SV for the single ventricle.

The time course of elastance is defined by the normalized elastance curve e(t) as follows:

3 $$e\left(t\right)=0.5\left[1-\text{cos}\left(\frac{\pi t}{{T}_{es,cc}}\right)\right] \left(0<t<2{T}_{es,cc}\right)$$

4 $$e\left(t\right)=0 \left(2{T}_{es,cc}<t<{T}_{c}\right)$$

where t is the time from the start of systole, T_es,cc_ is the duration of systole, and T_c_ is the duration of the cardiac cycle. Using e(t), the instantaneous pressure–volume relationship is described by [17]:

5 $$P_{{cc}} \left( t \right) = e\left( t \right) \cdot P_{{es,cc}} \left( t \right) + \left[ {1 - e\left( t \right)} \right] \cdot P_{{ed,cc}} \left( t \right)$$

The time advance from atrial systole to ventricular systole (DT) is calculated as a fixed fraction of T_c_ (DT = 0.02 T_c_). The function of each chamber is characterized by the parameters E_es,cc_, T_es,cc_, V_0,cc_, A_cc_, B_cc_ and e(t). Although the same e(t) is used for all chambers, the other parameters are different between ventricular and atrial chambers (Table 1).

**Table 1:** Baseline parameters used in the Fontan model

Diameter of fenestration, mm	2.5
Heart rate (HR), beats/min	80
Duration of cardiac cycle (T_c_), ms	750
Time advance of atrial systole, ms	15

	SV	SA
Time to end systole (T_es_), ms	200	120
End-systolic elastance (E_es_), mmHg/ml	13.1	1.64
Scaling factor of EDPVR (A), mmHg	1.15	0.197
Exponent for EDPVR (B), ml^−1^	0.0753	0.865
Unstressed volume (V_0_), ml	0	0

	Aortic	Atrioventricular
Valvular resistance (forward), mmHg s ml^−1^	0.001	0.001

	Systemic (s)	Pulmonary (p)
Arterial resistance (R_a_), mmHg s ml^−1^	1.5	0.075
Characteristic impedance (R_c_), mmHg s ml^−1^	0.0984	0.0655
Venous resistance (R_v_), mmHg s ml^−1^	0.0491	0.0492
Arterial capacitance (C_a_), ml/mmHg	0.61	3.97
Venous capacitance (C_v_), ml/mmHg	24.2	2.52

*SV* single ventricle, *SA* single atrium

### Vascular system

Pulmonary and systemic vascular systems are modeled as modified three-element Windkessel models. Each vascular system is modeled by lumped venous (C_v_) and arterial (C_a_) capacitances, a characteristic impedance (R_c_), a lumped arterial resistance (R_a_), and a resistance proximal to C_v_ (R_v_). Arterial and venous capacitors for systemic circulation are denoted by C_a,s_ and C_v,s_, respectively, and those for pulmonary circulation by C_a,p_ and C_v,p_ (Table 1). Impedance and resistances for systemic circulation are denoted by R_c,s_, R_a,s_ and R_v,s_ and those for pulmonary circulation by R_c,p_, R_a,p_ and R_v,p_. The relationship between pressure (P_c_) and volume (V_c_) in each capacitance C is described by the following linear formula:

6 $${P}_{c}=\frac{{V}_{c}}{C}$$

The changes in volume in each capacitance [dV(t)/dt] are described by the differential equations below.

7 $$\frac{dV\left(t\right)}{dt}=\sum {Q}_{inflow}\left(t\right)-\sum {Q}_{outflow}\left(t\right)$$

where Q_inflow_(t) and Q_outflow_(t) are volumetric inflow and outflow, respectively of each compartment. Each valve is described as an ideal diode connected serially to a small resistor (R_AV_; aortic, R_AVV_; atrioventricular).

### Fenestration

A simplified Bernoulli equation is used to simulate the relation between flow and pressure drop across the fenestration.

8 $$\Delta P=4{{f}_{v}}^{2}=4{\left(\frac{{Q}_{f}}{\pi {r}^{2}}\right)}^{2}=64\frac{{{Q}_{f}}^{2}}{{\pi }^{2}{D}^{4}}$$

where ΔP is the pressure gradient between central venous pressure and atrial pressure, and f_v_, Q_f_, r and D are flow velocity, volumetric flow, radius, and diameter of fenestration, respectively.

### Calculation of arterial and venous oxygen saturation

The single atrial O_2_ content was calculated as the sum of the O_2_ contents in the pulmonary veins and the blood from the fenestration. The O_2_ content in the blood from the fenestration was assumed to be equal to that in the systemic vein. Given that difference between arterial and venous O_2_ contents balances the whole body’s O_2_ consumption, the arterial and venous O_2_ saturations (SaO_2_ and SvO_2_, respectively) were calculated using the following equations for Q_p_, Q_s_ and Q_f_ (L/min):

9 $$SaO_{2} \times \left( {1.34 \times Hb \times 10 \times Q_{s} } \right)\; = \;S_{{PV}} O_{2} \times \left( {1.34 \times Hb \times 10 \times Q_{p} } \right) + SvO_{2} \times \left( {1.34 \times Hb \times 10 \times Q_{f} } \right)$$

10 $$CV{O}_{2}\times BSA=\left(1.34\times Hb\times 10\times {Q}_{s}\right)\times \left(Sa{O}_{2}-Sv{O}_{2}\right)$$

where S_pv_O_2_ is the pulmonary venous O_2_ saturation, CVO_2_ (ml O_2_ min^−1^ m^−2^) is the whole body O_2_ consumption, BSA (m^2^) is the body surface area, and Hb (g/dl) is the hemoglobin concentration. The constant 10 (dl/L) converts L to dl, and 1.34 (ml O_2_/g) converts hemoglobin content to oxygen content. The following assumptions are used in the calculation: S_pv_O_2_ = 0.99 (dimensionless), CVO_2_ = 185 ml O_2_ min^−1^ m^−2^, BSA = 0.58 m^2^, and Hb = 14.0 g/dl [18].

### Clinical data collection

Six patients who underwent the fenestrated Fontan operation at Okayama University Hospital between September 2017 and February 2021, and whose fenestration was patent one year after surgery were included in this study. The institutional review board of Okayama University Hospital approved the study (Institutional Review Board 2009-023; approval: October 23, 2020). Due to the observational nature of the study, the requirement for written informed consent was waived. The mean age at surgery was 33.5 ± 7.6 months. Five patients were male, and one was female. Five had hypoplastic left heart syndrome and one had single ventricle with common atrioventricular valve. There was no atrioventricular valve regurgitation in the six patients. Preoperative hemodynamic data were shown in Table 2.

**Table 2:** Hemodynamic data before the Fontan operation

BSA (m^2^)	0.45 ± 0.04
HR (bpm)	97 ± 14
SBP (mmHg)	73.3 ± 7.5
DBP (mmHg)	37.3 ± 5.9
CVP (mmHg)	9.8 ± 1.9
SpO_2_ (%)	84.5 ± 2.5
Q_p_/Q_s_	0.64 ± 0.06

Data were presented as mean values ± standard deviation

*BSA* body surface area, *HR* heart rate, *SBP* systolic blood pressure, *DBP* diastolic blood pressure, *CVP* central venous pressure, *SpO*_*2*_ oxygen saturation, *Q*_*p*_*/Q*_*s*_ pulmonary/systemic blood flow ratio

Clinical data for this study were collected one year after the Fontan operation. Cardiac catheterization and echocardiography data at one year after surgery included the diameter of fenestration (D), body surface area (BSA), heart rate (HR), blood pressure (BP), central venous pressure (CVP), oxygen saturation (SpO_2_), and pulmonary vascular resistance index (PVRI). The average data from these patients are presented in Table 3. The diameter of the fenestration was the effective diameter as measured by echocardiography, which was usually smaller than the creation diameter at surgery.

**Table 3:** Comparison between clinical and simulation data

	Clinical data (n = 6)	Simulation data
Diameter of fenestration (mm)	2.58 ± 0.38	2.5
BSA (m^2^)	0.58 ± 0.09	0.58
HR (bpm)	84 ± 17	80
SBP (mmHg)	87.0 ± 15.1	87.2
DBP (mmHg)	44.8 ± 7.6	44.2
CVP (mmHg)	11.8 ± 1.7	11.9
SpO_2_ (%)	91.2 ± 3.3	90.6
PVRI (Wood units m^2^)	1.97 ± 0.94^*^	1.83

Clinical data, presented as mean values ± standard deviation, were obtained from 6 patients 1 year after fenestrated Fontan operation

*BSA* body surface area, *HR* heart rate, *SBP* systolic blood pressure, *DBP* diastolic blood pressure, *CVP* central venous pressure, *SpO*_*2*_ oxygen saturation, *PVRI* pulmonary vascular resistance index

*One patient’s data was not available (n = 5)

### Protocols

Initially, the baseline state was simulated using the fenestrated Fontan model. The parameters for the baseline model are detailed in Table 1 [13, 19, 20]. We solved simultaneous differential Eqs. (1)–(10) using MATLAB/Simulink (MathWorks Inc., Natick, MA, USA). In this model, with a fixed HR of 80 bpm, BSA of 0.58 m^2^ and fenestration diameter of 2.5 mm, each parameter was fine-tuned to align simulation variables with clinical hemodynamic measurements, such as BP, CVP, SpO_2_, and PVRI as shown in Table 3.

Subsequently, to assess the impact of varying fenestration diameters, we adjusted the diameter from 0 to 4.5 mm in 0.5 mm increments while maintaining systolic BP at the same value of the baseline model by adjusting stressed blood volume (SBV). We then compared hemodynamic variables between the Fontan models with and without fenestration.

Finally, to evaluate the effects of fenestration on various cardiovascular dysfunctions, hemodynamic simulation was performed using the models with elevated pulmonary (I) and systemic vascular resistances (IV), as well as systolic (II) and diastolic ventricular dysfunctions (III). In these models, the diameter of fenestration was fixed at 2.5 mm.

I. To simulate elevated pulmonary vascular resistance (PVR), R_a,p_ was stepwisely increased from 0.0001 to 0.525 mmHg s ml^-1^ in increments of 0.075 mmHg s ml^-1^. As a result, PVR index [PVRI = (R_c,p_ + R_a,p_ + R_v,p_ )× BSA (mmHg s ml^-1^ m^2^) = 9.67 × R_a,p_ + 1.11 (Wood units m^2^)] increased from 1.11 to 6.18 Wood units m^2^.
II. To simulate systolic ventricular dysfunction, E_es_ was stepwisely decreased from 13.1 to 3.2 mmHg/ml in decrements of 3.3 mmHg/ml.
III. To simulate diastolic ventricular dysfunction, ventricular stiffness constant, B, was stepwisely increased from 0.075 to 0.120 /ml in increments of 0.015 /ml.
IV. To simulate elevated systemic vascular resistance (SVR), R_a,s_ was stepwisely increased from 0.8 to 5.0 mmHg s ml^-1^ in increments of 0.7 mmHg s ml^-1^. As a result, SVR index [SVRI = (R_c,s_ + R_a,s_ + R_v,s_ ) × BSA (mmHg s ml^-1^ m^2^)= 9.67 × R_a,s_ + 1.42 (Wood units m^2^)] increased from 9.16 to 49.8 Wood units m^2^.

In these models, systolic BP was maintained at the baseline model level by adjusting SBV. Diastolic BP, cardiac index (CI), CVP and SpO_2_ were calculated for each model.

## Results

### Baseline

The hemodynamic data derived from the computational simulation are presented in Table 3. The initial parameters were adjusted to align with the average hemodynamic data collected from six patients.

### Diameter of fenestration

**Fig. 3:**
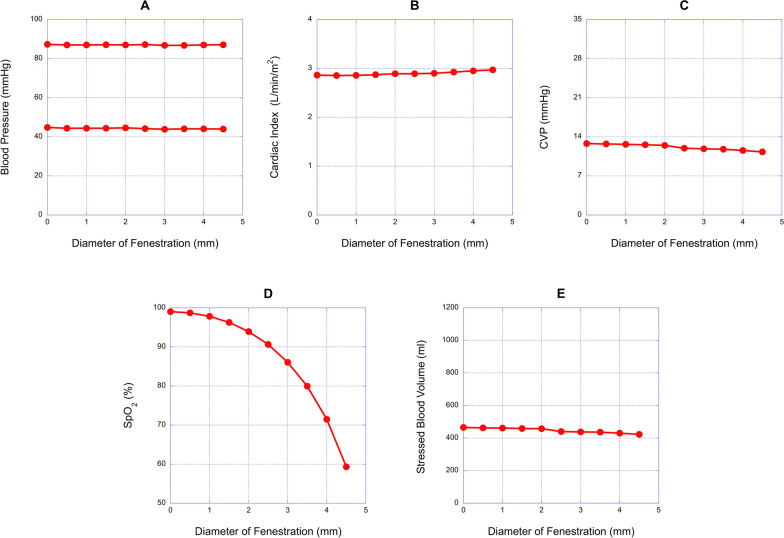
Relationships between fenestration diameters and hemodynamic variables. **A**, **B**, **C**, **D** and **E**, the relationships between diameter and blood pressure (BP), cardiac index (CI), central venous pressure (CVP), oxygen saturation (SpO_2_) and stressed blood volume (SBV), respectively

Figure 3 illustrates the impact of varying fenestration diameters on the hemodynamics of Fontan circulation. The fenestration diameter was adjusted from 0 to 4.5 mm, with all other parameters except SBV remaining constant. To maintain systolic BP at 87.2 mmHg, SBV was altered in increment of 1.0 ml (Fig. 3A and E). An increase in fenestration diameter resulted in a slight rise in CI (Fig. 3B). CVP in the model with a 4.0 mm fenestration was 0.4 mmHg lower than in the 2.5 mm fenestration model (11.5 vs 11.9 mmHg) (Fig. 3C). Conversely, an increase in fenestration diameter significantly reduced SpO_2_ from 90.6% at 2.5 mm to 71.5% at 4.0 mm (Fig. 3D).

### 2.5 mm fenestrated Fontan model vs Fontan model without fenestration

**Fig. 4:**
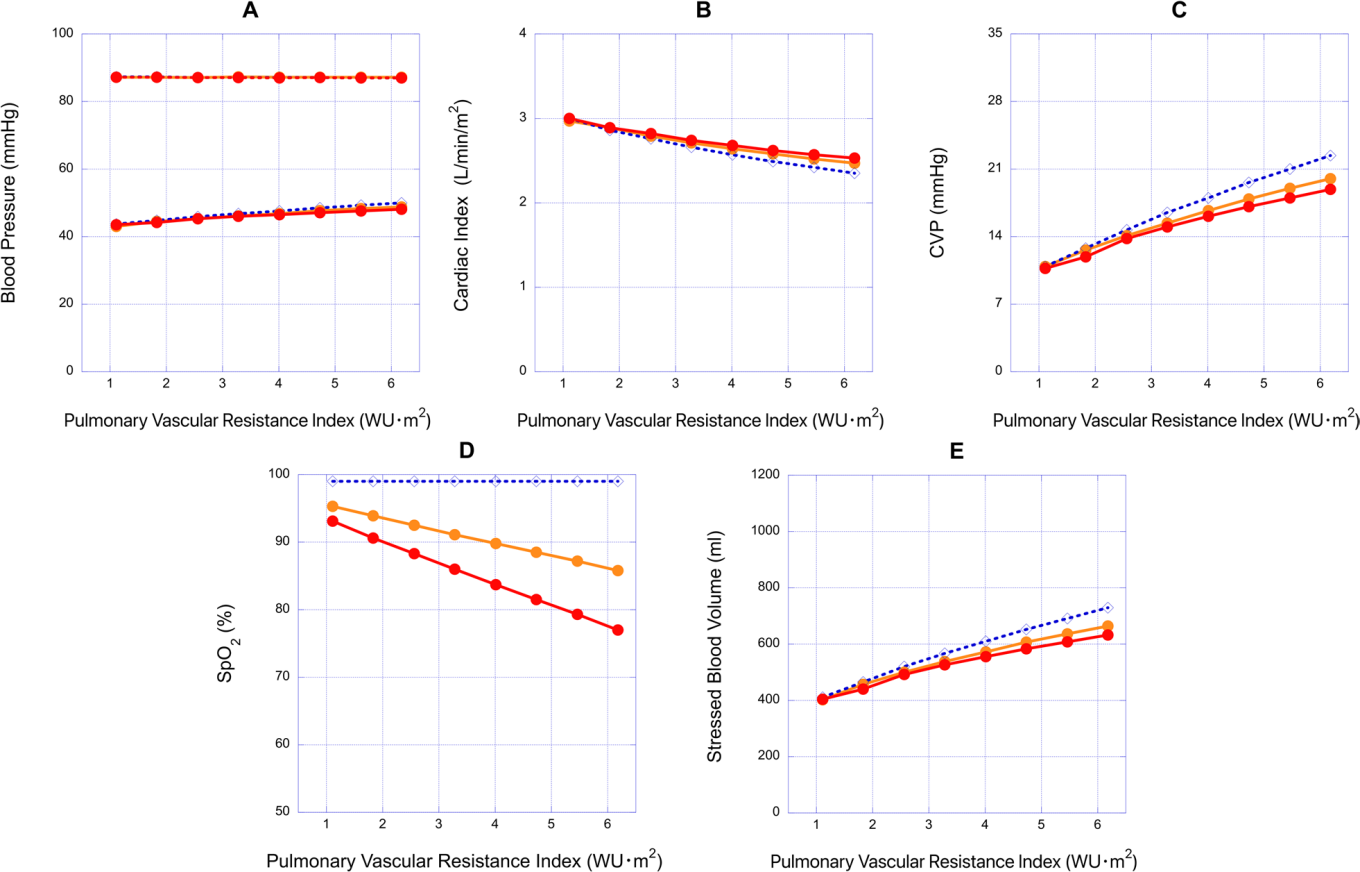
Relationships between pulmonary vascular resistance index (PVRI) and hemodynamic variables in the 2.0 mm and 2.5 mm fenestrated Fontan models and the Fontan model without fenestration. **A**, **B**, **C**, **D** and **E**: the relationships between PVRI and blood pressure (BP), cardiac index (CI), central venous pressure (CVP), oxygen saturation (SpO_2_) and stressed blood volume (SBV), respectively. Red circle, 2.5 mm fenestrated Fontan model; orange circle, 2.0 mm fenestrated Fontan model; blue diamond, Fontan model without fenestration

**Fig. 5:**
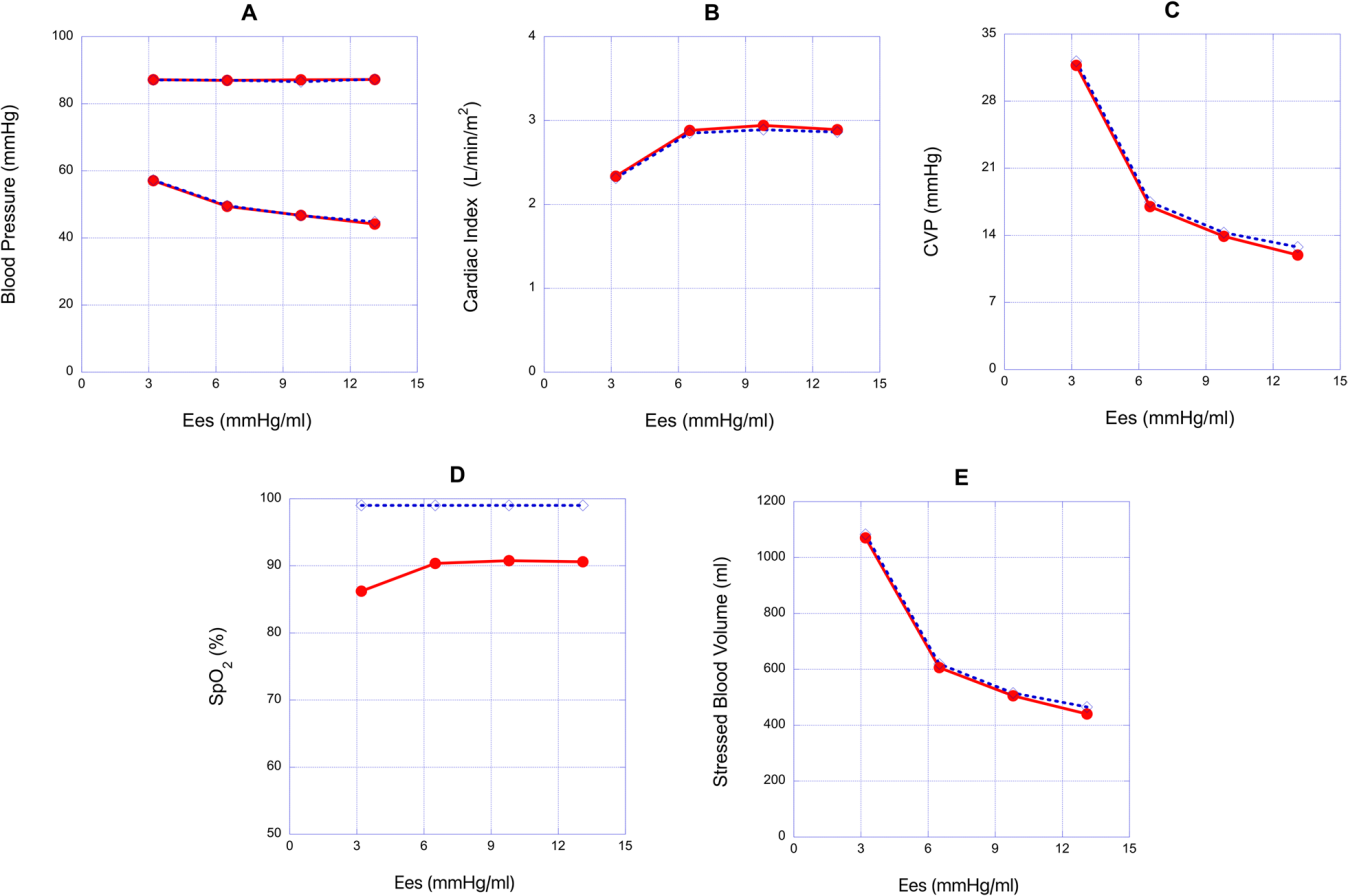
Relationships between end-systolic elastance (E_es_) and hemodynamic variables in the 2.5 mm fenestrated Fontan model and the Fontan model without fenestration. **A**, **B**, **C**, **D** and **E**: the relationships between E_es_ and blood pressure (BP), cardiac index (CI), central venous pressure (CVP), oxygen saturation (SpO_2_) and stressed blood volume (SBV), respectively. Red circle, 2.5 mm fenestrated Fontan model; blue diamond, Fontan model without fenestration

**Fig. 6:**
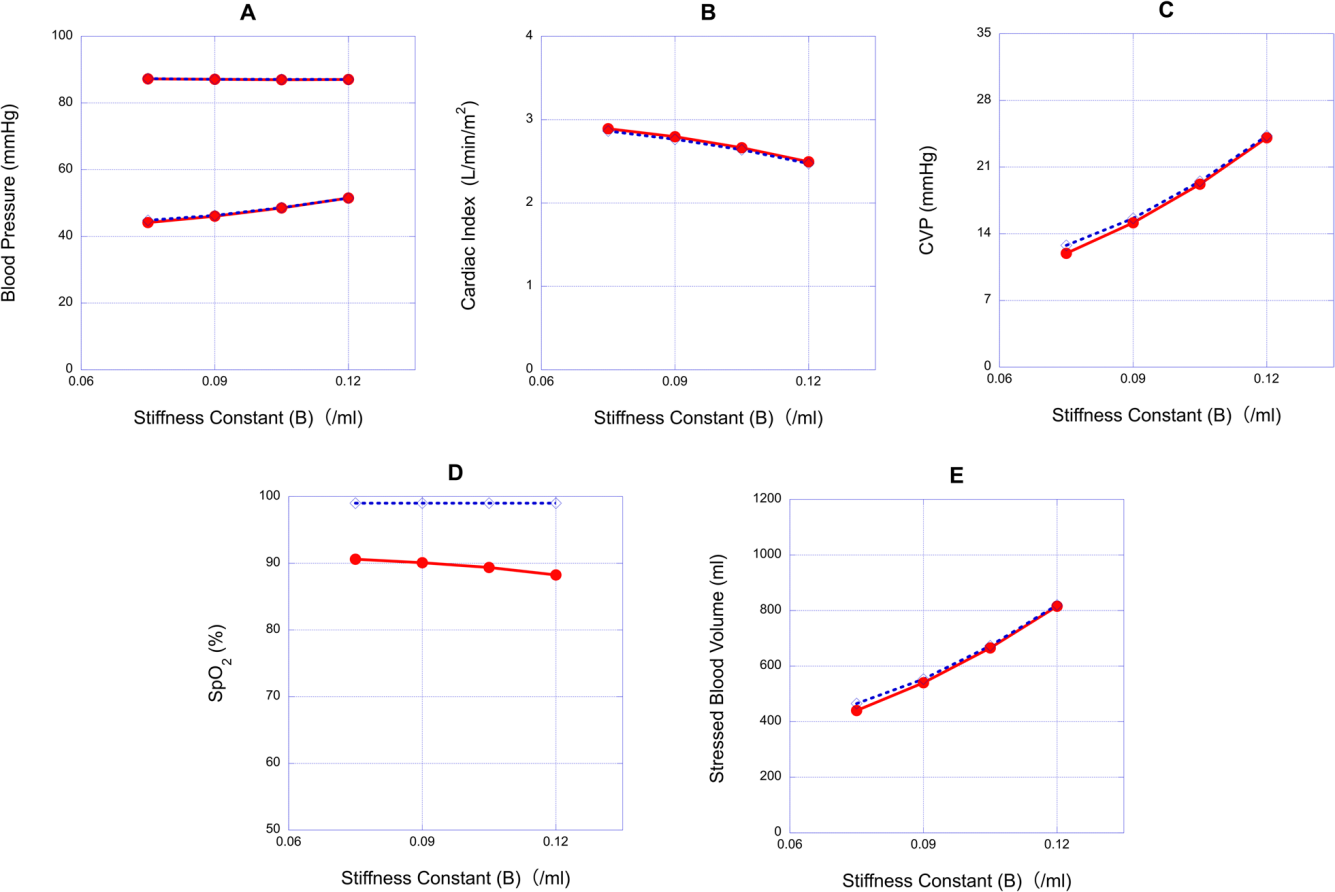
Relationships between ventricular stiffness constant (B) and hemodynamic variables in the 2.5 mm fenestrated Fontan model and the Fontan model without fenestration. **A**, **B**, **C**, **D** and **E**: the relationships between B and blood pressure (BP), cardiac index (CI), central venous pressure (CVP), oxygen saturation (SpO_2_) and stressed blood volume (SBV), respectively. Red circle, 2.5 mm fenestrated Fontan model; blue diamond, Fontan model without fenestration

**Fig. 7:**
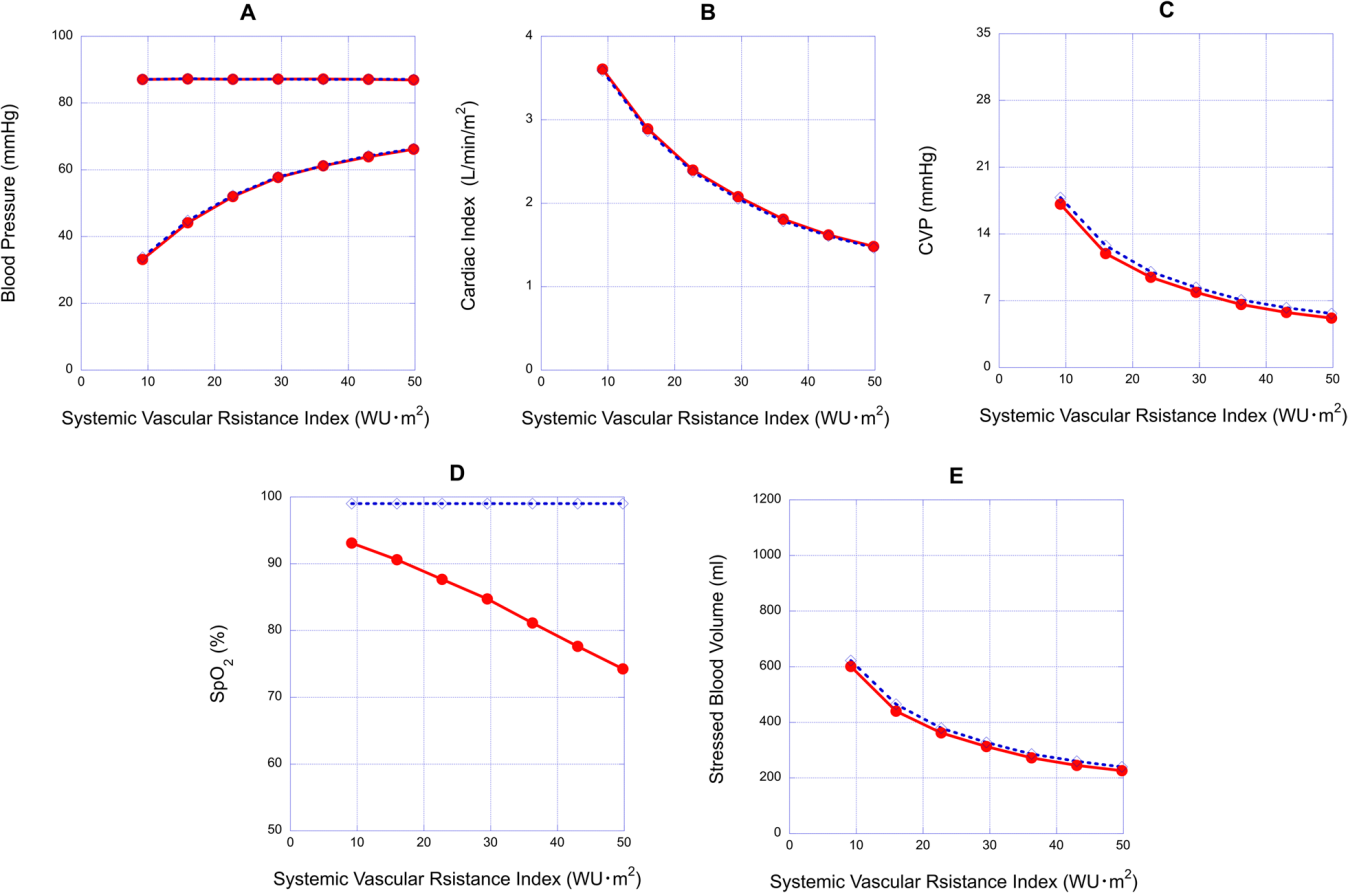
Relationships between systemic vascular resistance index (SVRI) and hemodynamic variables in the 2.5 mm fenestrated Fontan model and the Fontan model without fenestration. **A**, **B**, **C**, **D** and **E**: the relationships between SVRI and blood pressure (BP), cardiac index (CI), central venous pressure (CVP), oxygen saturation (SpO_2_) and stressed blood volume (SBV), respectively. Red circle, 2.5 mm fenestrated Fontan model; blue diamond, Fontan model without fenestration

The hemodynamic effects of a 2.5 mm fenestration were investigated by modifying various parameters, including the pulmonary vascular resistance index (PVRI), end-systolic elastance (E_es_), stiffness constant (B), and systemic vascular resistance index (SVRI). In both the 2.5 mm fenestrated Fontan model and the Fontan model without fenestration, systolic BP was maintained at 87.2 mmHg by adjusting the SBV in increments of 1 m1 (Figs. 4A, 5A, 6A and 7A).

In the model with elevated PVRI, fenestration significantly reduced CVP and SBV compared to the model without fenestration. Specifically, when PVRI was 3.28 Wood units m^2^, fenestration lowered CVP from 16.5 to 15.0 mmHg (Fig. 4C) and SBV from 567 to 526 ml (Fig. 4E). With PVRI at 4.01 Wood units m^2^, fenestration decreased CVP from 18.0 to 16.1 mmHg (Fig. 4C) and SBV from 610 to 555 ml (Fig. 4E). Conversely, fenestration significantly reduced SpO_2_ from 99.0 to 83.7% (Fig. 4D). Fenestration also slightly lowered diastolic BP and increased CI compared to the model without fenestration (Fig. 4A and B). When PVRI was 3.28 Wood units m^2^, fenestration raised CI from 2.66 to 2.74 L/min/m^2^ and decreased diastolic BP from 46.8 to 46.0 mmHg. With PVRI at 4.01 Wood units m^2^, fenestration increased CI from 2.57 to 2.68 L/min/m^2^ and decreased diastolic BP from 47.6 to 46.5 mmHg compared to the model without fenestration (Fig. 4A and B). In an additional simulation with 2.0 mm fenestration, the reduction in CVP and SBV was less than that with 2.5 mm fenestration, but SpO_2_ was higher in the 2.0 mm fenestration model (Fig. 4C, D and E).

In the models with reduced E_es_, elevated B or elevated SVRI, the effects of fenestration were limited. In such cases, fenestration rarely affected CI, diastolic BP, CVP, and SBV. When E_es_ was reduced from 13.1 to 3.2 mmHg/ml, fenestration slightly increased CI from 2.31 to 2.34 L/min/m^2^ (Fig. 5B) and decreased CVP from 32.2 to 31.7 mmHg (Fig. 5C) and SBV from 1083 to 1070 ml (Fig. 5E) compared to the model without fenestration. When B was elevated from 0.075 to 0.120/ml, fenestration slightly increased CI from 2.47 to 2.50 L/min/m^2^ (Fig. 6B) and decreased CVP from 24.3 to 24.1 mmHg (Fig. 6C) and SBV from 820 to 815 ml compared to the model without fenestration (Fig. 6E). When SVRI was increased from 15.9 to 36.2 Wood units m^2^, fenestration slightly increased CI from 1.78 to 1.81 L/min/m^2^ (Fig. 7B), decreased CVP from 7.09 to 6.61 mmHg (Fig. 7C) and decreased SBV from 286 to 272 ml compared to the model without fenestration (Fig. 7E). However, fenestration significantly decreased saturation from 99.0 to 86.2% at a reduced E_es_ of 3.2 mmHg/ml, 88.2% at an increased B of 0.120 /ml, and 81.1% at an increased SVRI of 36.2 Wood units m^2^ (Figs. 5D, 6D and 7D, respectively) compared to the model without fenestration.

## Discussion

Since the introduction of fenestration in Fontan operations in 1989 [6]**,** it has been recognized that creating a fenestration can lower central venous pressure and boost ventricular preload, leading to an increase in cardiac output. Various clinical studies have noted that fenestration contributes to reduced pleural effusions and, as a result, shorter hospital stays [8, 9]. While fenestration is deemed advantageous for high-risk patients, there are reports suggesting that it does not enhance long-term prognosis due to drawbacks such as desaturation and the risk of thromboembolism [7]. Consequently, the decision to implement fenestration is typically based on the policy of the institution or the discretion of the surgeon.

To determine the scenarios in which cases fenestration yields significant hemodynamic benefits, we modeled the cardiovascular system of the fenestrated Fontan circulation using a lumped parameter model. The primary advantage of this model is its ability to simulate hemodynamics quickly, even on a standard desktop computer. However, there have been few studies focusing on hemodynamic simulations of fenestrated Fontan circulation. Kurishima et al. developed a lumped parameter model for the fenestrated Fontan circulation, demonstrating that fenestration is most effective in the patients with good ventricular function and low PVR [14]. Ahmad et al. explored the impact of fenestration size, identifying the diameter at which the oxygen availability decreases significantly [15]. However, their models were not calibrated using data from actual Fontan patients but instead used baseline cardiovascular parameters from the literatures [11, 19, 21]. The parameters used in their models were based on healthy young adult subjects with a body weight of 75 kg and a body surface area of 1.9 m^2^. In our study, hemodynamic variables were adjusted to closely reflect the average clinical data from real Fontan patients. As a result, we demonstrated that fenestration effectively increased ventricular preload and improved hemodynamics in models with elevated PVR. Conversely, fenestration did not enhance hemodynamics in models with impaired cardiac function or elevated SVR.

This study indicates that fenestration can potentially reduce CVP and SBV in patients with elevated PVR compared to those without fenestration. These findings are consistent with several clinical studies where fenestration was shown to decrease CVP and increase cardiac output [6, 8]. It is generally believed that a larger fenestration leads to greater reductions in CVP and SBV. However, it also results in significant desaturation. This aligns with a previous simulation study showing that oxygen availability effectively decreased as fenestration size increased [15]. In our study, the fenestration diameter was varied, from 0 to 4.5 mm, with all other parameters except SBV remaining constant. In the model with 3 mm fenestration, CVP decreased from 12.8 to 11.9 mmHg and SBV from 465 to 438 ml compared to the model without fenestration. However, 3-mm fenestration resulted in SpO_2_ below 90% (86.0%). Moreover, larger fenestrations caused further reductions in SpO_2_ (80.0% at 3.5 mm, 71.5% at 4.0 mm and 59.3% at 4.5 mm). Therefore, in a clinical setting, determining the appropriate fenestration diameter, either before or during surgery, is crucial to avoid severe desaturation. Although adequate SpO_2_ after fenestration has yet to be determined, we believe that SpO_2_ above 90% is ideal and SpO_2_ of 85% is acceptable. Ko et al. recently reported that the median oxygen saturation in patients with fenestration was 89.0% (IQR: 86, 92.5) and that there was no significant difference in survival or freedom from Fontan failure between patients with and without fenestration [22].

In this study, we further explored the effects of a 2.5 mm fenestration by varying E_es_, B, SVRI and PVRI. Fenestration proved effective in reducing CVP and SBV while maintaining CI, specifically when varying PVRI. This reduction in SBV could offer advantages for perioperative management in Fontan patients, potentially leading to decreased perioperative volume infusion and subsequently a reduction in pleural effusions [8, 9]. However, a significant decrease in SpO_2_ was noted in our simulations. With a 2.5 mm fenestration, SpO_2_ decreased to 83.7% when PVRI was 4.01 Wood units m^2^. Although elevated PVR might indicate a need for fenestration, it is crucial to balance the fenestration diameter with an acceptable level of SpO_2_. Kurishima et al. suggested that the maximum of PVRI that can enhance hemodynamics without compromising SpO_2_ should be below 3 Wood units m^2^ [14]. Patient-specific hemodynamic simulations using the lumped parameter model may aid in determining the appropriate fenestration diameter.

In the model with impaired systolic/diastolic ventricular function or increased SVR, the benefits of fenestration, such as reduced CVP and SBV, disappear. In these models, fenestration only lowered SpO_2_, which became a disadvantage. Interestingly, when SVRI was varied (Fig. 7B), the cardiac indices of the models with and without fenestration decreased with increasing SVRI. This suggests the importance of systemic vasodilative therapy in Fontan patients. Because systemic vasoconstriction causes significant desaturation in fenestrated Fontan patients, systemic vasodilative therapy may be more important than in Fontan patients without fenestration.

The criteria for the Fontan procedure have evolved due to advancements in surgical techniques, improved patient selection, and enhanced perioperative management [2, 23, 24]. Several studies suggest that fenestration should be considered for high-risk patients rather than being performed routinely [25, 26]. Key risk factors for Fontan failure include elevated PVR, impaired cardiac function, and valvular regurgitation. Our study has shown that fenestration notably improves hemodynamics in patients with elevated PVR. Therefore, when evaluating the necessity for fenestration in Fontan operation, it is crucial to determine if the patient has elevated PVR. In real clinical practice, it is also essential to consider mixed conditions such as cardiac dysfunction combined with pulmonary hypertension. Consequently, patient-specific hemodynamic simulations could be invaluable in deciding the need for fenestration and selecting the appropriate fenestration diameter. On the other hand, physical activity can dramatically alter hemodynamics after Fontan operation with fenestration by altering several parameters, including heart rate, ventricular elastance, vascular resistances and capacitances, and stressed blood volume. Because our steady-state simulation may not predict hemodynamic changes during exercise, the development of a dynamic model that incorporates parameter interaction will be helpful in predicting postoperative hemodynamics during exercise.

## Limitation

First, the parameters utilized in this study were not patient-specific. While adjustments were made to align with the average values from six fenestrated Fontan patients, individualized simulation might be required for accurate hemodynamic evaluations due to significant variability in baseline parameters. Moreover, the clinical data employed in this analysis were gathered one year after the fenestrated Fontan operation, which could limit the study’s findings as parameters in pediatric patients evolve over time.

Second, the study’s methodology involved altering only one parameter at a time, keeping the others constant. However, in actual clinical scenarios, a change in one parameter, like pulmonary vascular resistance, could influence the others. To incorporate the parameter interaction into the simulation, a method is needed that can identify the parameter changes from the patient’s real-time hemodynamic data. Therefore, further investigation is needed to make our simulation more realistic.

Third, valvular regurgitation was not simulated in this study. Valvular regurgitation, especially atrioventricular regurgitation, can affect the simulation results. To evaluate patient-specific hemodynamics, it is necessary to include valvular regurgitation in the simulation model.

Fourth, intrapulmonary shunts and collateral blood vessels were excluded in the present model. Therefore, SpO_2_ was equivalent to S_pv_O_2_ in the model without fenestration. However, in the clinical setting, the presence of intrapulmonary shunts may decrease SpO_2_ even in Fontan patients without fenestration. To evaluate the effect of intrapulmonary shunts, another model with intrapulmonary shunts is needed.

## Conclusion

Our simulation study reveals that fenestrated Fontan operation significantly reduces CVP and SBV in the model with elevated PVR. However, in models with systolic dysfunction (reduced E_es_), diastolic dysfunction (elevated stiffness constant) or elevated SVR, fenestration has little advantages on hemodynamics but decreases oxygen saturation. Therefore, fenestration may effectively improve hemodynamics only in the patients with elevated PVR.

## Data Availability

The datasets used and analyzed during the current study are available from the corresponding author on reasonable request.

## Abbreviations

B: Stiffness constant
BP: Blood pressure
BSA: Body surface area
C_a,p_: Pulmonary arterial capacitance
C_a,s_: Systemic arterial capacitance
CI: Cardiac index
CO: Cardiac output
CVO_2_: Whole body O_2_ consumption
CVP: Central venous pressure
C_v,p_: Pulmonary venous capacitance
C_v,s_: Systemic venous capacitance
D: Diameter of fenestration
E_es_: End-systolic elastance
Hb: Hemoglobin concentration
HR: Heart rate
IQR: Interquantile range
PVR: Pulmonary vascular resistance
PVRI: Pulmonary vascular resistance index
R_c,p_: Characteristic impedance of pulmonary artery
R_c,s_: Characteristic impedance of systemic artery
R_a,p_: Pulmonary arterial resistance
R_a,s_: Systemic arterial resistance
R_AV_: Aortic valvular resistance
R_AVV_: Atrioventricular valvular resistance
R_v,p_: Pulmonary venous resistance
R_v,s_: Systemic venous resistance
SaO_2_: Arterial O_2_ saturation
SAP: Single atrial pressure
SBV: Stressed blood volume
SpO_2_: Oxygen saturation
S_PV_O_2_: Pulmonary venous O_2_ saturation
SvO_2_: Systemic venous O_2_ saturation
SVR: Systemic vascular resistance
SVRI: Systemic vascular resistance index
